# The co-circulating transmission dynamics of SARS-CoV-2 Alpha and Eta variants in Nigeria: A retrospective modeling study of COVID-19

**DOI:** 10.7189/jogh.11.05028

**Published:** 2021-12-25

**Authors:** Shi Zhao, Salihu S Musa, Marc KC Chong, Jinjun Ran, Mohammad Javanbakht, Lefei Han, Kai Wang, Nafiu Hussaini, Abdulrazaq G Habib, Maggie H Wang, Daihai He

**Affiliations:** 1 JC School of Public Health and Primary Care, Chinese University of Hong Kong, Hong Kong, China; 2 CUHK Shenzhen Research Institute, Shenzhen, China; 3 Department of Applied Mathematics, The Hong Kong Polytechnic University, Hong Kong, China; 4 Department of Mathematics, Kano University of Science and Technology, Wudil, Nigeria; 5 School of Public Health, Shanghai Jiao Tong University School of Medicine, Shanghai, China; 6 Nephrology and Urology Research Center, Baqiyatallah University of Medical Sciences, Tehran, Iran; 7 School of Global Health, Chinese Center for Tropical Diseases Research, Shanghai Jiao Tong University School of Medicine, Shanghai, China; 8 Department of Medical Engineering and Technology, Xinjiang Medical University, Urumqi, China; 9 Department of Mathematical Sciences, Bayero University, Kano, Nigeria; 10 Department of Medicine, Bayero University, Kano, Nigeria

## Abstract

**Background:**

The COVID-19 pandemic poses serious threats to public health globally, and the emerging mutations in SARS-CoV-2 genomes has become one of the major challenges of disease control. In the second epidemic wave in Nigeria, the roles of co-circulating SARS-CoV-2 Alpha (ie, B.1.1.7) and Eta (ie, B.1.525) variants in contributing to the epidemiological outcomes were of public health concerns for investigation.

**Methods:**

We developed a mathematical model to capture the transmission dynamics of different types of strains in Nigeria. By fitting to the national-wide COVID-19 surveillance data, the transmission advantages of SARS-CoV-2 variants were estimated by likelihood-based inference framework.

**Results:**

The reproduction numbers were estimated to decrease steadily from 1.5 to 0.8 in the second epidemic wave. In December 2020, when both Alpha and Eta variants were at low prevalent levels, their transmission advantages (against the wild type) were estimated at 1.51 (95% credible intervals (CrI) = 1.48, 1.54), and 1.56 (95% CrI = 1.54, 1.59), respectively. In January 2021, when the original variants almost vanished, we estimated a weak but significant transmission advantage of Eta against Alpha variants with 1.14 (95% CrI = 1.11, 1.16).

**Conclusions:**

Our findings suggested evidence of the transmission advantages for both Alpha and Eta variants, of which Eta appeared slightly more infectious than Alpha. We highlighted the critical importance of COVID-19 control measures in mitigating the outbreak size and relaxing the burdens to health care systems in Nigeria.

The coronavirus disease 2019 (COVID-19), whose etiological agent is the severe acute respiratory syndrome coronavirus 2 (SARS-CoV-2), posed serious threat to global health and the pandemic is still ongoing. As of July 30, 2021, around 200 million COVID-19 cases have been reported with over 4 million associated deaths globally [1]. However, the evolving mutations of SARS-CoV-2 has continuously changed the infectiousness profiles and clinical severity of COVID-19, which challenged the campaign against the pandemic [2,3]. These mutations usually have higher infectivity [4,5], and may typically establish their transmission dominance at population scale [6-8], which is one of the determinants of infectious diseases outbreaks [2].

By the end of 2020, the SARS-CoV-2 strains carrying novel genetic mutations in the ‘spike’ and other regions were detected and started to circulate in Nigeria. The SARS-CoV-2 mutants were later recognized as Alpha (ie, B.1.1.7) variants, and Eta (ie, B.1.525) variants. The Alpha variants that carried N501Y amino acid substitution were first detected in the United Kingdom (UK) [9], and then spread elsewhere globally, eg, Brazil [10] and the US [11]. In recognizing the increasing risks of transmission and hospitalization [2,12-15], Alpha variants were classified as variants of concern (VoC) by the World Health Organization (WHO). The Eta variants was first detected in Nigeria and the UK [16]. Although it appeared less impactful than the threats from Alpha variants at the global scale, the Eta variants subsequently became dominant in Nigeria [2] and were classified as variants of interest (VoI) by WHO.

The timing of emergence of both Alpha and Eta variants roughly coincided with the occurrence of the second major epidemic wave in Nigeria. The rapid growth of both variants coincided with increasing incidences of COVID-19 cases, which are suspected as a sign of selection advantage. Although the transmission advantage has been found for Alpha variants [12-15], the risk of transmission remains largely unassessed for Eta variants and in the settings of African regions. Given the co-circulation of both Alpha and Eta variants and the wild type, the transmission dynamics may be stratified by different types of strains, which is also of interest for investigation.

In this work, the co-circulating transmission dynamics of Alpha and Eta variants were modelled and compared to assess risks of transmission for SARS-CoV-2 variants. Our analysis enables us to provide epidemiological insights into the competing and transmission processes in viruses co-circulation context.

## METHODS

### Data and study period

This is a retrospective modelling study using time series data sets. The COVID-19 surveillance data of daily number of new cases in Nigeria were collected via the World Health Organization (WHO) coronavirus (COVID-19) dashboard [1]. The SARS-CoV-2 sequences in Nigeria were obtained from the Global Initiative on Sharing All Influenza Data (GISAID) platform [17], the sampling distributions of which were reported on a weekly basis.

**Figure 1:**
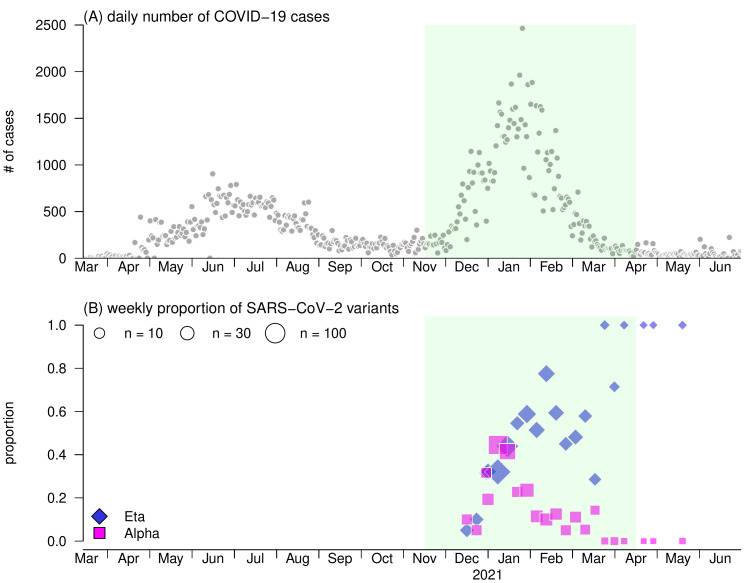
The COVID-19 cases and proportions of SARS-CoV-2 variants in Nigeria. **Panel A.** The daily number of COVID-19 cases. **Panel B.** Weekly proportions of Alpha (ie, B.1.1.7) and Eta (ie, B.1.525) SARS-CoV-2 variants in Nigeria. In Panel B, the size of dots indicates the sample size of strains in each week. In both panels, the shading green area highlights the study period from November 16, 2020 to April 15, 2021, when the second major epidemic wave of COVID-19 occurred in Nigeria. In axis labels, ‘#’ denotes the word ‘number’.

Due to the emergency of Eta and Alpha variants by the end of 2020 in Nigeria, the second epidemic wave occurred, and eventually ended before May 2021 (**Figure 1**). We consider the study period covered the course of the second epidemic, which was from November 16, 2020 to April 15, 2021. During this period, there were 98 763 COVID-19 cases reported, and 615 SARS-CoV-2 sequences collected, of which the sampling coverage was 0.62% per reported case. Among these 615 strain samples, 140 and 254 were Alpha and Eta variants, respectively, and the rest 221 samples were grouped as the original strains that were circulating before Alpha or Eta variants. The classification of Alpha and Eta variants was conducted by the GISAID platform, which referred to the defining genetic mutations of each variant.

### Epidemic model

To capture the co-circulating transmission dynamics of COVID-19 in Nigeria, we formulate the classic three-strain susceptible-exposed-infectious-removed (SEIR) model as an ordinary differential equation system in Eqn (1).


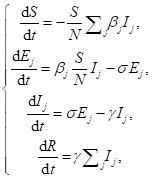
 for *j* = 1, 2, and 3.(1)

**Figure 2:**
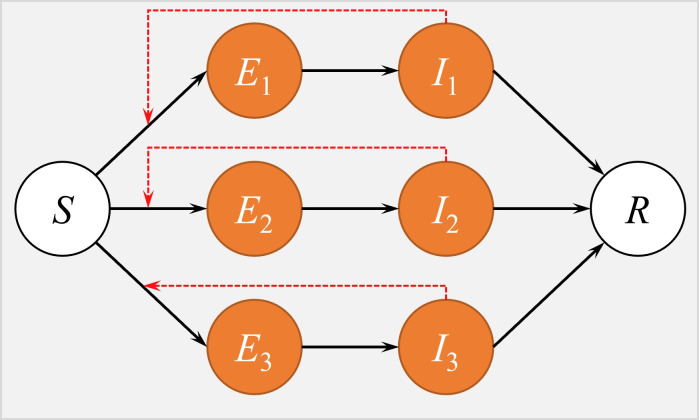
The schematic diagram of epidemic model in Eqn (1). The classes in red indicate individuals are infected by SARS-CoV-2. The back bold arrows are transition paths, and the red dashed arrows are transmission paths. The compartments *S*, *E*, *I*, and *R* indicated susceptible, exposed, infectious, and removed classes in the epidemic model.

Here, the subscripts ‘1’, ‘2’ and ‘3′ denote the model classes or parameters that are relevant to original strain, Alpha and Eta variants, respectively. The parameter *β* is the transmission rate. The parameter *σ* is the transition rate from *E* to *I*, the reciprocal of which (*σ*^−1^) is the mean latent period. The parameter *γ* is the removing rate, the reciprocal of which (*γ*^−1^) is the mean infectious period. Since d*N*/d*t* = 0, the total population size (N = *S* + *E*_1_ + *E*_2_ + *E*_3_ + *I*_1_ + *I*_2_ + *I*_3_ + *R*) is a constant. The schematic diagram of Eqn (1) was illustrated in **Figure 2**.

### Reproduction number and transmission advantage

The basic reproduction number is the expected number of cases directly generated by one typical case during the infectious period in a wholly susceptible population [18]. Using the next generation matrix approach [19], the basic reproduction number of cases infected by the *j*-th type of strains is

*R_j_* = *β_j_/γ*, for *j* = 1, 2, and 3, (2)

which are in line with [20,21]. Hence, the basic reproduction number *R*_0_ of the whole system in Eqn (1) is the weighted average as follows:


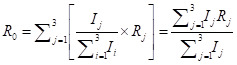
.

Epidemiologically, the outbreak is likely to occur with number of cases increasing when reproduction number is larger than 1 [22], and vice versa. As a well-studied metric that considers both reproducibility and survivability of the seed case, reproduction number is typically adopted to measure the fitness of a pathogen in maintaining its transmission [23].

For a mutated strain, its multiplicative transmission advantage (*η*) against another (eg, its recent ancestor) is typically quantified by the ratio between two fitness [24], namely relative fitness. Thus, the transmission advantage *η_i,j_* of the *i*-th type against the *j*-th type of strains is defined in Eqn (3):

*η_i,j_* = *R_i_/R_j_*, (3)

which was also adopted to study the transmission dynamics of influenza [25], and COVID-19 [2,7,14,15]. If *η_i,j_* > 1, the *i*-th type strains are more transmissible than the *j*-th type strains, and vice versa. Specifically, we are interested in comparing the transmission advantaged of Alpha and Eta variants against the original strains or each other, ie, *η*_2,1_, *η*_3,1_, and *η*_3,2_.

### Statistical inference

#### Initial settings and fixed parameters

As the first outbreak of COVID-19 in human history, we assume that [*S* (*t* = 0) / *N* =] 98% of population was susceptible at the start of simulation, ie, *t* = 0. Since the first report of Alpha and Eta variants was around November or December in Nigeria, we mimic the situation that the new strains started emerging from low prevalent levels. As such, we consider that the 108 COVID-19 cases reported on November 15, 2020 were composed by 106, 1, and 1 cases infected by the original strains, Alpha and Eta variants, respectively. The remaining proportion of population was assigned to removed class *R*.

The total population in Nigeria is assumed at *N* = 202 million individuals. For the model parameters in Eqn (1), the mean latent period is set at *σ*^−1^ = 3.3 days referring to [26,27], and the mean infectious period is set at *γ*^−1^ = 3.2 days referring to [27-29].

#### Likelihood framework

By adopting likelihood frameworks, we linked the theoretical outcomes from model simulation to the real-world observations from COVID-19 surveillance. The measurement noises from the observatory process were accounted for by using the following likelihood functions.

For the daily number of new cases, a negative binomial (NB) distributed likelihood function was formulated in Eqn (4), which followed previous frameworks in [30,31].

*c*(*t*) ~ NB(mean = *r* × *z*(*t*), dispersion = *k*), (4)

where

*z*(*t*) =  *∫*_day_ *_t_* *σ* (*E*_1_ + *E*_2_ + *E*_3_) d*t*.

Here, *c*(*t*) denotes the reported (or observed) number of COVID-19 cases, and *z*(*t*) denotes the theoretical number of SARS-CoV-2 infections on day *t*. Note that *z*(*t*) accounted for both symptomatic and asymptomatic infections. The dispersion parameter *k* in the NB distribution accounted for the superspreading potentials of COVID-19, and *k* is fixed at 0.43 referring to previous estimates [32-34]. The term *r* denotes the reporting (or ascertainment) ratio, which considers the ascertainment efforts of SARS-CoV-2 infections, and thus we have 0<*r*<1. Alternatively, the NB distribution in Eqn (4) can be simplified as a Poisson distribution, which was adopted in [25,35,36], and this change will not affect main conclusions.

For different types of SARS-CoV-2 strains, a multinomial distributed likelihood function was formulated in Eqn (5):

{*x_j_*(*t*)} ~ multinomial (size = Σ*_j_x_j_*(*t*), probabilities = {*p_j_*(*t*)}), for *j* = 1, 2, and 3, (5)

where {*x_j_*(*t*)} is the vector of numbers of the *j*-th type strains observed at time *t*. For the probabilities vector {*p_j_*(*t*)}), we have

*p_j_*(*t*) = (*∫*_day_ *_t_* *σE_j_* d*t*)/*z*(*t*), for *j* = 1, 2, and 3,

and Σ*_j_p_j_*(*t*) = 1 strictly holds for all time *t*.

To construct the overall likelihood, we calculated the product of the likelihood functions defined in Eqns (4) and (5).

#### Fitting and estimating scheme

The model in Eqn (1) was simulated stochastically for 151 days corresponding to the time interval between November 16, 2020 and April 15, 2021. The systematic noise from the epidemic model was accounted for by using the Euler’s fix-time-step multinomial method with d*t* = 1/365.25 year [30,31,37], which is equivalent to 1 day at the scale of 1 year.

To account for the temporal changes in COVID-19 transmissibility, the reproduction numbers were estimated on a time-varying basis. We estimate the time-varying values of *R*_1_, *R*_2_, and *R*_3_ as step functions for 5 consecutive periods including from November 16, 2020 to December 15, 2020, …, from March 16, 2021 to April 15, 2021. Given the values of reproduction numbers, the value of *β* can be calculated according to Eqn (2) backwardly. The COVID-19 case ascertainment ratio (*r*) was modelled as a fixed parameter to be estimated for 3 types of strains.

We adopted a Bayesian fitting procedure with Metropolis-Hastings Markov chain Monte Carlo (MCMC) algorithm with noninformative prior distributions. Based on the likelihood function, the MCMC is conducted with 5 chains and 100000 iterations for each chain, including 40000 as for the burn-in period, to obtain the posterior estimates. The convergence of each MCMC chain was checked by using the trace plot and Gelman-Rubin-Brrooks convergence diagnostic. The median estimate and 95% credible intervals (95% CrI) are calculated. The fitting and estimating procedures were carried out by using **R** statistical software (version 3.5.1), and no specific package was adopted.

For the sensitivity analysis, we repeated the fitting procedures with *σ*^−1^ at 2.3 or 4.3 days and *γ*^−1^ at 2.2 or 4.2 days, and re-checked the consistency and significance of model estimates. Since the exact date of variants emerging were untraceable, we also checked the sensitivity of simulation by slightly changing the initial settings (ie, timing, and number) of seed cases, which merely affected the model outcomes at minor scales.

## RESULTS

For the second epidemic wave in Nigeria (**Figure 1A**), the epidemic curve started increasing by the end of November 2020, reached the peak with size at nearly 2000 COVID-19 cases per day in January 2021, and gradually decreased until a constant low scale after March 2021. By replacing the original strains circulating in Nigeria, both Alpha and Eta SARS-CoV-2 variants emerged and increased roughly by the end of 2020, and reached a total of more than 90% proportion since January 2021 (**Figure 1B**). After January 2021, the proportion of Eta variants increased and gradually reach fixation in April 2021, whereas the proportion of Eta variants decreased towards 0 at the same time.

**Figure 3:**
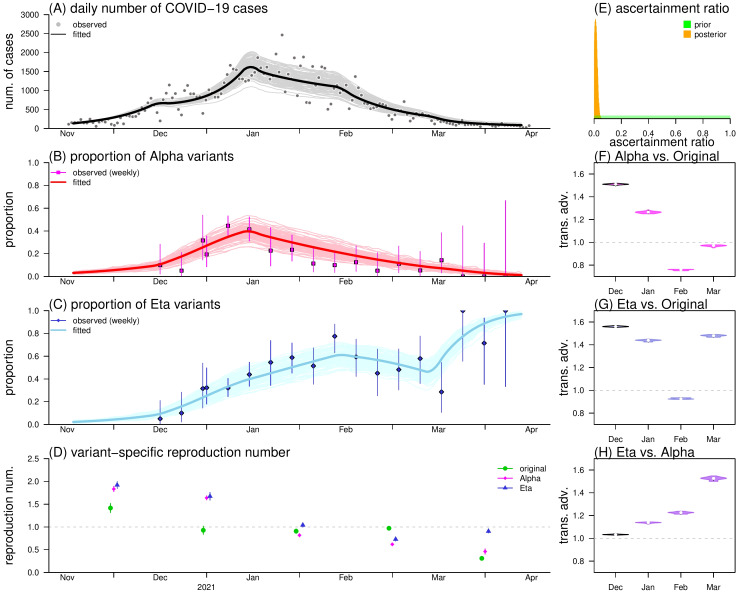
The simulation outcomes. **Panel A.** The fitting and estimating results including daily number of COVID-19 cases. **Panel B.** The proportions of the Alpha SARS-CoV-2 variant. **Panel C.** The proportion of the Eta SARS-CoV-2 variant. **Panel D.** The estimated reproduction numbers of each type of strains. **Panel E.** The case ascertainment ratio. **Panels F-H.** The transmission advantages estimates of *η*_2,1_, *η*_3,1_, and *η*_3,2_. In panels A-C, dots are the observations, and curves are fitting outcomes, among which bord curves are the median estimates and curves with light colors are 100 times realization of model simulation. In panels B and C, the vertical bars are the 95% Jeffreys intervals of strain proportions calculated empirically. In panel D, the dots and bars are the median estimates and their 95% CrIs, respectively. Panel E shows the prior (green) and posterior (orange) distributions of the case ascertainment ratio (*r*). Panels F, G, and H show the posterior distributions of *η*_2,1_, *η*_3,1_, and *η*_3,2_, respectively.

We reported that the simulation outcomes had a satisfactory fitting performance to the observations of COVID-19 cases (**Figure 3****,** Panel A), and SARS-CoV-2 variant proportions (**Figure 3****,** Panels B and C). The estimated reproduction numbers decreased steadily from 1.5 to 0.8 (**Figure 3****,** Panel D), and appeared larger than 1 before 2021, which were consistent with the growing phase of the epidemic curve. The overall decreasing trends of reproduction number was considered as a consequence of the implementation of disease control measures in Nigeria [38]. In **Figure 3****,** Panel E, we estimated the case ascertainment ratio (*r*) at 1.03% (95% CrI = 0.86, 1.49), which implied from 3% to 6% of the whole population in Nigeria were infected by SARS-CoV-2 during the second epidemic wave. The underreporting of COVID-19 cases was also speculated in Nigeria previously [39]. By checking the sensitivity of analyses, we reported that the model estimates are consistent with the main results (data not shown).

On average, the Alpha variants were estimated 14% more infectious than the original strains during the entire period of the second epidemic wave in Nigeria. This digit was estimated at 44% for Eta variants against original strains, and 26% for Eta variants against Alpha variants. In December 2020, transmission advantages of Alpha and Eta variants were estimated at *η*_2,1_ = 1.51 (95% CrI = 1.48, 1.54), and *η*_3,1_ = 1.56 (95% CrI = 1.54, 1.59), respectively (**Figure 3****,** Panels F and G**)**. In January 2021, we estimated the transmission advantages of Eta against Alpha variants *η*_3,2_ = 1.14 (95% CrI = 1.11, 1.16) (**Figure 3****,** Panel H).

## DISCUSSION

The study showed the time-varying transmission advantages of genetic variants. When both Alpha and Eta variants were competing against the original strains in December 2020, their transmission advantages were estimated at *η*_2,1_ = 1.51 (95% CrI = 1.48, 1.54), and *η*_3,1_ = 1.56 (95% CrI: 1.54, 1.59), respectively. Consistently, previous studies also reported a significant selection advantage in Alpha variants against the wild types (ie, *η*_2,1_) at similar scales [2,13-15,40]. In January 2021, when the original variants almost vanished, the competition became restrained between Alpha and Eta variants (**Figure 1****,** Panel B). We estimated *η*_2,1_ = 1.14 (95% CrI = 1.11, 1.16), and reported a weak but significant selection advantage of Eta against Alpha variants, which appeared in line with [2]. The reproduction number was estimated around 1.5 in December 2020, which appeared slightly lower than the basic reproduction number estimates in early 2020 [28,35,41,42]. This difference was probably because the reproduction number estimated in this study was subject to the impacts of non-pharmaceutical interventions (NPIs), eg, social distancing, city lockdown, and behavioral factors against long-term pandemic [38], which may lead to temporal changes in effective transmission advantage [12,14]. Nevertheless, we consider the estimates of transmission advantages in the growing phase of the epidemic approximated to their intrinsic values, when NPIs were not yet implemented or have less impacts.

Since both Alpha and Eta variants have relatively strong transmission advantage against the original strains, they became dominant rapidly within 2 months since emergence. It is worth noting that the trends of Alpha and Eta variants are roughly synchronized by the end of 2020 (**Figure 1**, panels B-C). Given that *η*_2,1_ and *η*_3,1_ were estimated at a similar scale, the competitions of Alpha and Eta variants against the original strains may be equally intensive during the initial stage of the second epidemic wave. The slight advantage in transmission of Eta against Alpha variants may explain the inflection point for the trend of Alpha variants (**Figure 1**, panel B), as well as the general increasing trend of Eta variants (**Figure 1**, panel C), which reached the fixation eventually. After all, the increased transmissibility of both variants might undermine the balance between the COVID-19 epidemic and implemented control measures during the first epidemic wave, and thus might contribute to the second epidemic wave in Nigeria.

Despite the epidemiological situations assessed within the study period, we discuss the updated situation in Nigeria by extending the period to date. Although Eta variants already reached fixation as of March 2021, their dominant position in Nigeria was challenged by the invasion of more infectious Delta (ie, B.1.617.2) variants since June 2021, which were first detected and circulated in India [43]. The circulation of Eta variants was surpassed, and thus vanished due to a much stronger selection advantage of Delta variants [2]. It was believed that Delta variants were the major cause of the ongoing third epidemic wave in Nigeria, and similar situations also occur in many other regions [2,8,43]. The transmission dynamics of Delta variants in Nigeria remain largely uninvestigated as the third epidemic wave is still ongoing. Regarding the threats from SARS-CoV-2 variants carrying novel genetic mutations, increasing numbers of studies reported that the neutralizing antibody activities from prior infection or vaccination scaled down against these variants [44-46], which imply increase in the risks of re-infection and breakthrough infection [47]. Higher risks of infection and clinical severities may lead to the increasing volumes of COVID-19 patients with critical conditions [4,7,8,40,48]. Although Eta variants have been almost fully replaced by Delta variants in Nigeria, they are still circulating elsewhere, and need future efforts for real-time monitoring and risk assessment. As such, the re-enforcement of COVID-19 control measures, eg, NPI and vaccination campaigns, becomes critically important to mitigate the outbreak size and to relax the burdens to health care systems in Nigeria and other places globally.

### Limitations

This study has the following limitations regarding the data sets, the assumptions and formulation of the epidemic model, and the interpretation of findings, which were partly pointed out in previous studies [6,13,49]. First, our analysis was based on the SARS-CoV-2 sequences data released in GISAID platform, and thus was subject to the selection bias of strain samples reported to the public domain [13]. Second, the impact of COVID-19 vaccination campaign in reducing the susceptibility was ignored from the epidemic model because only less than 0.1% of the population in Nigeria received two doses before May 2021. Third, consider the different settings and epidemiological situations across different regions in Nigeria, the local transmissibility of Alpha or Eta variants might be higher in the places where more variants circulated or more cases occurred [12-14]. We remarked that homogeneous mixing was assumed in Eqn (1), and thus our model cannot capture this potential spatial heterogeneity, which requires region-specified data sets and patchy framework in the model. Fourth, the likelihood formulation in Eqns (4) and (5) holds when COVID-19 cases and SARS-CoV-2 strains match along the same timeline. We consider the reporting delays of cases and strains data exists in close scale, and thus the effects of two reporting lags may be counteracted. Furthermore, with detailed reporting lag information of each individual case, the adjustment for reporting delay can be performed based on the current analytical framework. Fifth, the model outcomes rely on the fixed settings of *σ*^−1^ = 3.3 days and *γ*^−1^ = 3.2 days, which follow previous studies [26-29]. In the real-world situation, the values of *σ*^−1^ or *γ*^−1^ might be time-varying. However, the overall trends of the reproduction numbers are unlikely changed by a slight variation in *σ*^−1^ or *γ*^−1^, or similar, serial interval [36]. Thus, we neglect the impact of this limitation on the inference of variant-specific change in transmissibility, and our model can be extended to a more complex time-varying context with the information of the evolution in model settings. Sixth, the values of *σ*^−1^ or *γ*^−1^ might change for mutated variants theoretically. However, by screening the literature, we detect little evidence that *σ*^−1^ or *γ*^−1^ is varied associated with the Alpha or Eta variants, and thus fixed values were adopted for simplicity. Following the classic theory, the generation interval is the summation of *σ*^−1^ and *γ*^−1^ [50]. We remark that if *σ*^−1^ or *γ*^−1^ becomes smaller for Alpha or Eta variants, the transmission advantage estimated in this study will become larger than the real value. Given the model estimates consistently hold in sensitivity analyses, we remark that this limitation may be minor. Seventh, similarly, the case ascertainment ratio (*r*) was also considered as a fixed parameter for infections of all types of strains, and throughout the study period. The value of *r* is related to the changes in reporting guideline and ascertainment efforts, eg, testing capacity and contact tracing intensity. For different types of variants, the reporting of cases might be biased towards those with more detectable clinical conditions. Due to lack of these relevant information, our analysis is limited by a fixed case ascertainment ratio. Eighth, one of the simplifications was simultaneous emergence of the two mutants were considered in the epidemic model rather than sequential emergence. Although lack of detailed information, it was suspected that Alpha variants started increasing when Nigerians were returning from the UK for the Christmas. Our framework can be extended to model the sequential emergence if the exact importation (or emerging) dates of each variant were known. Although a delay in the seeding date will lead to a slight increase in the reproduction estimates, we remark these changes are unlikely to be major given the study period is relatively longer than the adjustments in seeding date. Ninth, the impact of re-infection is ignored in our model because the re-infection events of Alpha and Eta variants occurred at a relatively rarely chance comparing to the primary infection. Tenth, this study focuses on exploring the effects on changing the transmission dynamics associated with the co-circulating Alpha and Eta variants. However, the intrinsic biological mechanisms are commonly more complex and remain uncovered. Future studies are needed for exploring the relationship of how the mutations in SARS-CoV-2 affect the infectiousness profiles of COVID-19. Lastly, due to the lack of individual patients’ information, time-series data was used in this work, which means information loss during the data aggregation. With an ecological setting, our findings cannot guarantee causality, which needs to be verified by further biomedical experiments in more sophisticated contexts.

## CONCLUSION

The reproduction numbers in Nigeria were estimated to decrease from 1.5 to 0.8 in the second epidemic wave from November 2020 to April 2021. Our findings suggested evidence of the transmission advantages for both Alpha and Eta variants, of which Eta appeared slightly more infectious than Alpha. We highlighted the critical importance of COVID-19 control measures in mitigating the outbreak size and relaxing the burdens to health care systems in Nigeria.
